# Brunner's Gland Hyperplasia: A Massive Duodenal Lesion

**DOI:** 10.7759/cureus.7542

**Published:** 2020-04-04

**Authors:** Sundus Bhatti, Mohammed Alghamdi, Endashaw Omer

**Affiliations:** 1 Internal Medicine, University of Louisville School of Medicine, Louisville, USA; 2 Pathology, University of Louisville School of Medicine, Louisville, USA; 3 Gastroenterology, University of Louisville, Louisville, USA

**Keywords:** brunneroma, brunner's gland hyperplasia, iron deficiency anemia, gastric outlet obstruction

## Abstract

A 57-year-old male with a history of gastroesophageal reflux disease and esophageal strictures presented with melena and abdominal pain. He underwent an esophagogastroduodenoscopy, which revealed a 5-cm duodenal bulb mass causing partial obstruction of the gastric outlet. Endoscopic ultrasound showed a 5-cm, hypoechoic lesion, arising from the mucosal layer, with a large blood vessel feeding the lesion. Biopsy revealed benign Brunner’s gland hyperplasia. The large mass was causing symptomatic obstruction of the pylorus and iron deficiency anemia, and had risk for malignant transformation. Due to its size it was not amenable to endoscopic removal. Subsequently, he underwent exploratory laparotomy with pyloroplasty, duodenotomy and partial duodenal resection. Surgical pathology showed Brunner’s gland hyperplasia and was negative for malignancy.

## Introduction

The small intestine constitutes 75% of the gastrointestinal tract; however, small intestine tumors are extremely rare (5%), with duodenal tumors being more common than jejunal and ileal tumors [1]. Brunner’s glands are alkaline-fluid secreting duodenal glands in the submucosa of proximal duodenum. Brunner’s gland hyperplasia is a rare entity that accounts for 10% of all benign duodenal tumors, with an estimated incidence of 0.008% [2]. Brunner’s gland hyperplasia has an equivalent gender distribution and typically presents in the fifth or sixth decade of life [1]. Literature search shows reports of Brunner’s gland hyperplasia ranging in size from 0.7 to 12 cm (mean 4 cm), but there are few reported that are larger than 5 cm [1,3-7]. Brunner’s gland hyperplasia is usually an incidental finding during endoscopy or imaging studies, and endoscopy is the mainstay of management [8]. 

We present a case of Brunner's gland hyperplasia leading to a 5-cm symptomatic mass that required surgical resection due to its size. This case was presented as a poster and published as an abstract [9]. 

## Case presentation

A 57-year-old male with myasthenia gravis, gastroesophageal reflux disease (GERD) and esophageal strictures requiring dilations presented with a few weeks of melena and abdominal pain. His labs were notable for iron deficiency anemia: hemoglobin 9.4, iron 27, ferritin 7. He had been on pantoprazole 40 mg daily and was started on iron supplementation.

He underwent an esophagogastroduodenoscopy (EGD) and colonoscopy. EGD showed chronic duodenitis and a large, benign appearing, polypoidal mass with a large stalk and normal overlying mucosa, measuring about 4 cm, in the proximal duodenal bulb. This mass was causing partial obstruction of the gastric outlet. Biopsies of this mass were benign in pathology and negative for *Helicobacter pylori.* Colonoscopy showed benign colon mucosa with three hyperplastic polyps and diverticulosis. 

**Figure 1:**
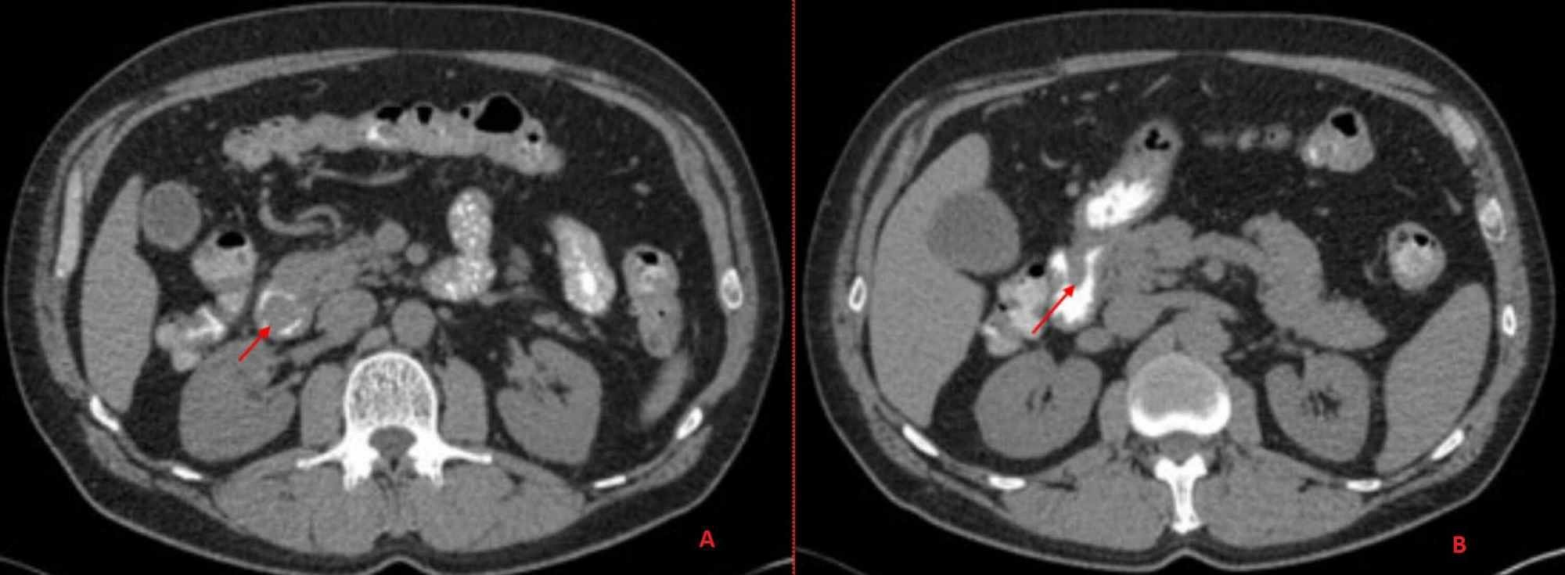
A CT abdomen with contrast shows an elongated, well-circumscribed, intraluminal mass, arising from the duodenal bulb, measuring 5.3 x 1.7 x 1.5 cm.

He was referred for an EGD with endoscopic ultrasound (EUS) for further evaluation of the mass, which was delayed by seven months as the patient had relocated. After establishing care with a gastroenterologist, a CT abdomen with contrast was performed which showed an elongated, well-circumscribed, intraluminal mass, arising from the proximal duodenal bulb, measuring approximately 5.3 x 1.7 x 1.5 cm, without evidence of lymphadenopathy or metastasis (Figure 1).

**Figure 2:**
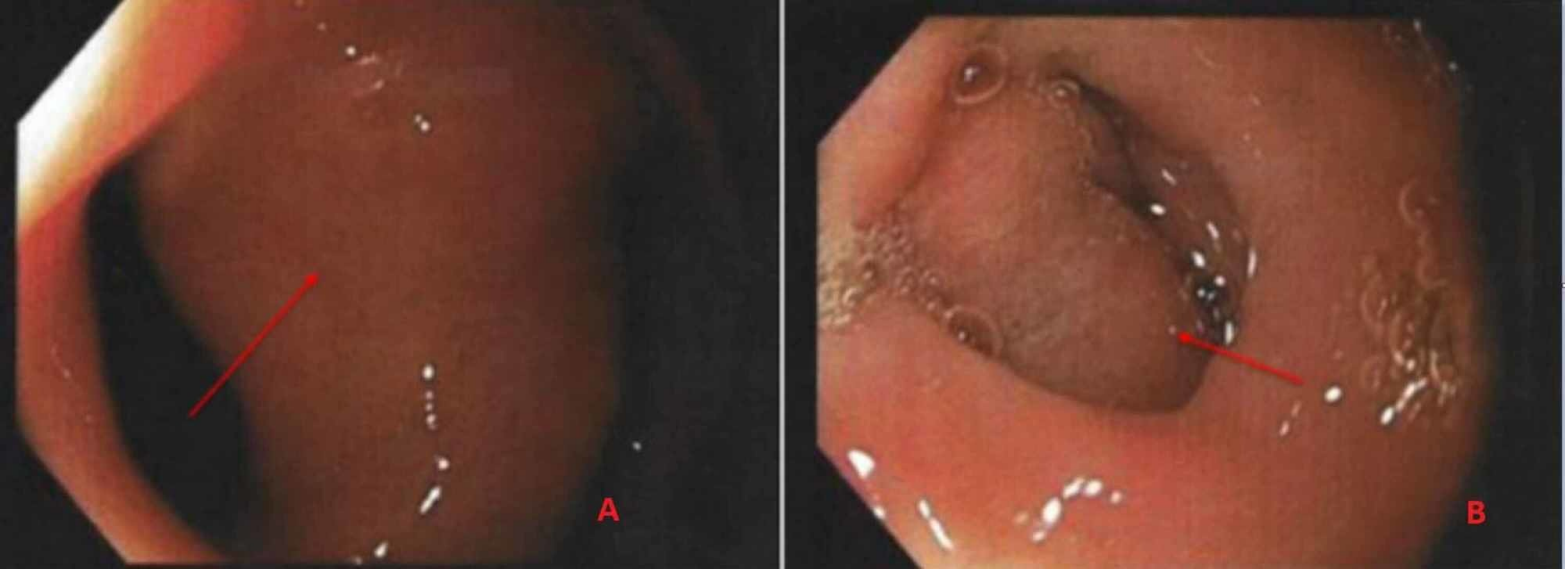
Esophagogastroduodenoscopy shows a large 5-cm polypoidal mass with a stalk in the duodenal bulb.

Repeat EGD showed a 5-cm polypoidal mass and a thick stalk in the proximal duodenal bulb (Figure 2), causing ball-valve like obstruction of the pyloric channel. The mucosal pattern of the lesion did not appear to be distinct from that of the duodenum. It had shallow surface ulcerations. Multiple biopsies were taken from the lesion.

**Figure 3:**
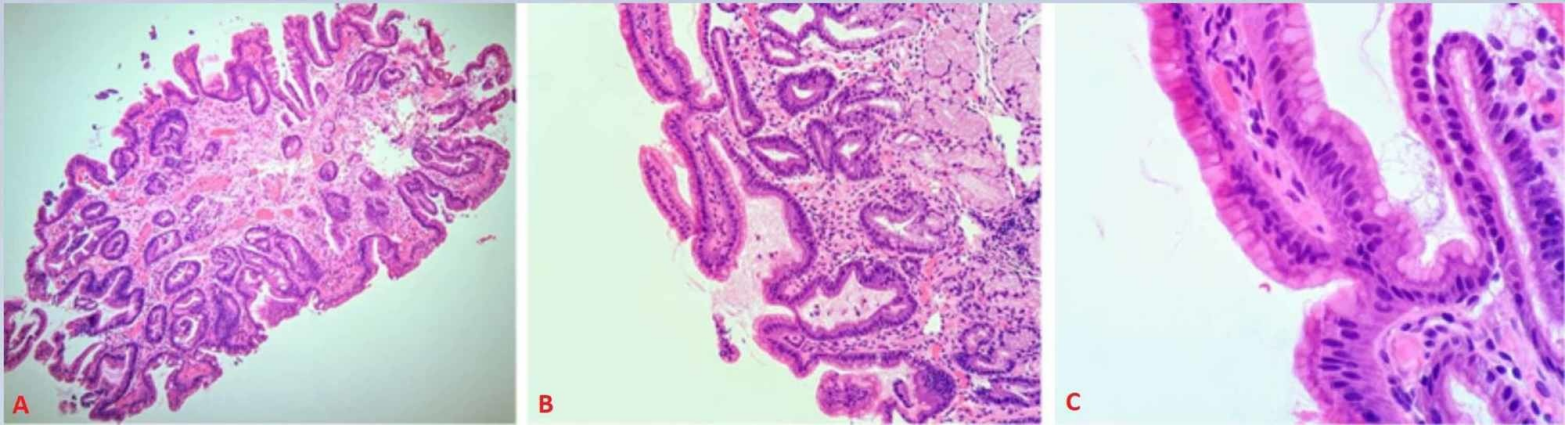
Histologic sections (hematoxylin and eosin stained, A; 10X, B; 20X, C; 40X) show duodenal mucosa with reactive changes and foveolar metaplasia.

Differential diagnosis at the time included tubular adenoma and carcinoid. Endoscopic biopsy revealed benign histology with foveolar cell metaplasia and reactive cellular changes. No adenomatous changes or malignancy were identified (Figure 3).

**Figure 4:**
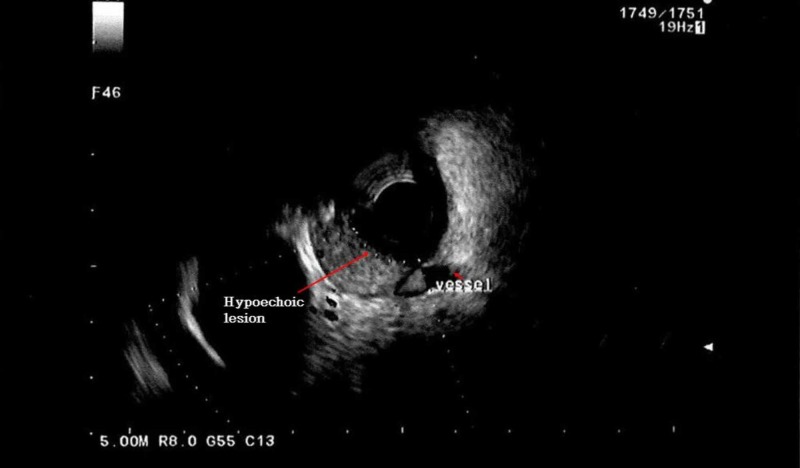
Endoscopic ultrasound shows a hypoechoic lesion arising from the mucosal layer, with a large blood vessel feeding the lesion.

EUS showed a hypoechoic lesion arising from the mucosal layer, with a large blood vessel feeding the lesion (Figure 4).

Due to its large size, the lesion was not amenable to endoscopic removal. He was referred for surgical removal of the mass, as it was causing iron deficiency anemia and symptomatic obstruction of the pylorus, and had risk for malignant transformation. He underwent exploratory laparotomy with pyloroplasty, duodenotomy and partial duodenal resection due to the lesion being symptomatic and concern that this may have malignant potential. 

**Figure 5:**
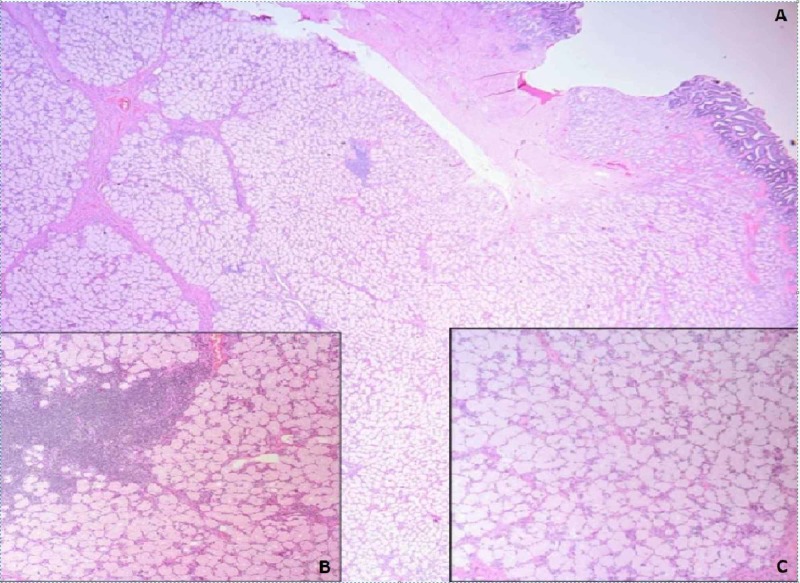
Histologic section A (hematoxylin and eosin, 10X) shows marked Brunner's gland hyperplasia in relation to the intact duodenal surface. Inset photomicrographs (hematoxylin and eosin, 40X) show the focal non-specific lymphocytic infiltrate (B) and benign glandular elements of this lesion (C).

Histological examination of the resection on routine hematoxylin and eosin slides showed Brunner’s gland hyperplasia and was negative for malignancy (Figure 5). 

He has been doing well postoperatively and reports resolution of his symptoms. 

## Discussion

The American Institute of Radiologic Pathology uses the terms “Brunner's gland hyperplasia” for lesions <5 mm in size and “Brunner’s gland hamartoma” for lesions >5 mm in size [10]. The underlying etiology of Brunner's gland hyperplasia is postulated to be secondary to excessive acid secretion, *Helicobacter pylori* infection or inflammation stimulating the Brunner's gland cells to produce alkaline secretions and subsequently undergoing hyperplasia [1]. 

Brunner’s gland hyperplasia is usually an incidental finding during endoscopy or imaging studies [5]. Symptomatic presentations include anemia, gastrointestinal bleeding, duodenal/ampullary obstruction and intussusception [10]. Literature search shows reports of Brunner’s hyperplasia ranging in size from 0.7 to 12 cm (mean 4 cm), but there are few reported that are larger than 5 cm, including one measuring 12 cm [1,3-7]. Although largely benign, Brunner’s gland hyperplasia has risk for malignant transformation [11]. 

Diagnosis requires histological examination of the mass, usually by endoscopy with excision or biopsy. CT scan and EUS can demonstrate the submucosal origin of the mass [12]. Mode of excision depends on the size, location and presentation of the lesion. Upper gastrointestinal endoscopy with polypectomy is preferred for smaller lesions. Laparoscopic polypectomy is also an option. Laparotomy is reserved for large, sessile tumors, failure of endoscopic approach or unstable bleeding patients [13]. Few cases reported in literature have been managed with laparotomy [5,8,14]. 

The potential cause of our patient’s large Brunner’s gland mass could be gastric acid hypersecretion given his severe GERD with peptic esophageal strictures. 

## Conclusions

Brunner’s gland hyperplasia, though often an incidental finding, can present symptomatically with larger sized masses. Although largely benign, there is rare potential for malignant transformation. The potential cause of our patient’s large Brunner’s gland mass could be gastric acid hypersecretion given his severe GERD with peptic esophageal strictures. 
